# Benzodiazepine receipt in adults with psychogenic non-epileptic seizures in the USA

**DOI:** 10.1136/bmjno-2024-000767

**Published:** 2024-09-18

**Authors:** Kevin Young Xu, Fábio A Nascimento, Binx Yezhe Lin, Tae Woo Park, Donovan T Maust, Hillary Samples, Greta A Bushnell

**Affiliations:** 1 Department of Psychiatry, Washington University School of Medicine in Saint Louis, St Louis, Missouri, USA; 2 Department of Neurology, Washington University School of Medicine in Saint Louis, St Louis, Missouri, USA; 3 Department of Psychiatry and Behavioral Sciences, Weill Institute for Neurosciences, UCSF, San Francisco, California, USA; 4 Department of Psychiatry, University of Pittsburgh School of Medicine, Pittsburgh, Pennsylvania, USA; 5 Department of Psychiatry, University of Michigan Medical School, Ann Arbor, Michigan, USA; 6 Center for Pharmacoepidemiology and Treatment Science, Rutgers Institute for Health, Health Care Policy and Aging Research, Rutgers University, New Brunswick, New Jersey, USA

**Keywords:** EPILEPSY, PSYCHIATRY, EPIDEMIOLOGY

## Abstract

**Background:**

Characterising benzodiazepine (BZD) prescribing to individuals with psychogenic non-epileptic seizures (PNES) is important for optimising PNES outcomes, but existing data is lacking.

**Methods:**

Using a nationwide administrative claims database (2016–2022), incident PNES was defined as an International classification of diseases, tenth revision, clinical modification (ICD-10-CM) diagnosis in an inpatient or outpatient healthcare encounter after a 1-year period with no documented diagnosis. We described clinical characteristics of adults with incident PNES and estimated the prevalence of outpatient BZD treatment in the baseline year and 30-day follow-up period, with secondary analyses stratifying by baseline ES, anxiety and/or insomnia diagnoses, representing common indications for BZD receipt. We used logistic regression to evaluate predictors of post-PNES BZD receipt.

**Results:**

Among 20 848 adults with incident PNES diagnosis, 33.1% and 15.1% received BZDs in the year and month prior to PNES diagnosis, respectively, and 18.1% received BZDs in the month following a PNES diagnosis; 5.4% of those without prior BZD prescriptions received BZDs after diagnosis. The median days’ supply was 30 days, with clonazepam, alprazolam and lorazepam representing the most common BZDs prescribed after PNES. Most people who received BZDs in the month prior to PNES diagnosis remained on BZDs in the month after PNES diagnosis (62.9%), with similar findings in the subcohorts without ES, anxiety and/or insomnia. Baseline BZD receipt and anxiety disorders, but not baseline ES diagnoses, were strong independent predictors of post-PNES BZD receipt.

**Conclusions:**

While new BZD initiation is rare after PNES, most individuals with BZD scripts 1 month before PNES continue scripts after diagnosis.

**WHAT IS ALREADY KNOWN ON THIS TOPIC**

- Benzodiazepines (BZD) may contribute to morbidity and confound clinical assessment of people with psychogenic non-epileptic seizures (PNES). Characterising current BZD prescribing to individuals with PNES is important for optimising their treatment and outcomes, but existing data is lacking.

**WHAT THIS STUDY ADDS**

- In an analysis of nationwide administrative data in the USA, we found that most of those on BZD treatment prior to PNES diagnosis continued BZD treatment after a new PNES diagnosis. Few adults newly initiated BZD treatment after a PNES diagnosis

**HOW THIS STUDY MIGHT AFFECT RESEARCH, PRACTICE OR POLICY**

- Over 60% of patients who were prescribed BZDs in the month prior to a new PNES diagnosis continued to receive outpatient BZDs in the month after the PNES diagnosis. Future research is needed to understand longitudinal outcomes associated with BZD exposure in people with PNES.

## Background

 A diagnosis of psychogenic non-epileptic seizures (PNES) is a stigmatised condition that frequently co-presents in people being evaluated for epileptic seizures (ES).^1^,^3^ PNES is difficult to differentiate from ES both clinically and via electroencephalogram.^1 4^ As a result, nearly one-third of patients referred to epilepsy care are suspected to have PNES by neurologists, with many concurrently or previously diagnosed with ES.^5 6^ The challenge of accurately diagnosing and treating PNES is underscored by the International League Against Epilepsy’s consensus clinical practice statements ranking PNES as among the top three neuropsychiatric problems associated with epilepsy.^7^

Because antiseizure drugs are not known to effectively treat PNES,^3^ there is concern that treating PNES as if it were ES may result in medically unnecessary diagnostic evaluations and acute care admissions, which contribute to a reduced quality of life in people with PNES.^3 8^ When considering the potential for iatrogenic harm in people with PNES,^5^ benzodiazepines (BZDs) merit particular concern.^9^ One chart review study found that BZD prescriptions were initiated in >70% of PNES episodes.^10^ While BZDs are commonly used both acutely and chronically for the treatment of ES, BZD use is associated with an increased risk of respiratory depression,^11^ addiction, cognitive impairment, drug-related poisoning and falls, accidents and injuries.^12^ Studies have also found prescription pain medications such as opioids to be prevalent among people with PNES and ES,^13^ raising concerns for the co-prescribing of opioids and BZDs, a risk factor for overdose death.^14 15^ Finally, although BZDs are commonly used to treat psychiatric conditions that are prevalent in adults with PNES, they can also complicate the differential diagnosis associated with ES. BZD withdrawal has clinical sequelae that may lead to seizures as well as anxiety, depersonalisation, depersonalisation and other symptoms that can mimic both PNES and ES,^12 16 17^ which may potentially lead to people being misdiagnosed with PNES that is actually BZD withdrawal.

Given the potential risks of BZD treatment, particularly in people with PNES, understanding current BZD prescribing to people with PNES is important to optimise their treatment outcomes. Yet, large-scale real-world data are lacking. Past studies on BZD prescribing in PNES are limited by relatively small sample sizes from select institutions rather than nationwide data, which restricts the ability to generalise across sites with wide variations in care protocols.^9 10^ To our knowledge, no studies have used nationwide administrative claim data in the USA to characterise adults diagnosed with PNES and outpatient BZD prescribing surrounding PNES diagnoses. Therefore, using a nationwide sample of privately insured US adults, we aimed to characterise individuals with a new PNES diagnosis and examine BZD prescription prior to and following the PNES diagnosis. We further aimed to examine the length and type of BZD prescriptions immediately following a PNES diagnosis to better understand prescribing decisions. For a secondary aim, we examined BZD receipt in those with and without epilepsy and other common BZD indications.

## Methods

### Study overview

The study cohort was identified from the US Merative MarketScan commercial claims and encounters database from 1 January 2016 to 31 December 2022, which contains patient-level data on insurance enrolment and demographics, outpatient filled prescription medications and inpatient and outpatient healthcare encounters. This analysis was reviewed by the Rutgers University Institutional Review Board and determined to be exempt. The Strengthening the Reporting of Observational Studies in Epidemiology and the Reporting of Studies Conducted Using Observational Routinely Collected Health Data Statement for Pharmacoepidemiology reporting guidelines were followed.

### Participants

The study cohort included adults aged 18–64 years newly diagnosed with PNES from 2016 to 2022. New PNES diagnosis (‘index diagnosis’) was defined as the first diagnosis in the data with at least 1 year without a prior PNES diagnosis. Diagnoses for PNES were identified as an International classification of diseases, tenth revision, clinical modification (ICD-10-CM) code for F44.5 occurring in any service setting, including inpatient admissions, emergency department encounters and/or ambulatory outpatient visits. The positive predictive value of PNES diagnostic codes was 83.3% in other populations.^18^ We required continuous insurance enrolment in the year before the index PNES diagnosis to ensure we could capture new PNES diagnoses and collect covariates. We additionally required continuous insurance enrolment in the 30 days after the index diagnosis to examine follow-up BZD prescriptions. As a secondary analysis, we compared BZD prescriptions in people with PNES who did and did not have an ICD-10 diagnosis for ES, anxiety and/or sleep-related disorders. While some patients with ES will also experience non-epileptic events,^19^ we assumed that a baseline diagnosis of ES, anxiety and/or sleep-related disorders could potentially suggest a greater burden of past BZD receipt. We stratified individuals by the history of epilepsy diagnosis in the 1-year baseline period preceding the index diagnosis of PNES. This was ascertained via the presence of >1 ICD-10 code for G40, made by a clinician during a healthcare encounter in the year preceding PNES diagnosis, across any service setting. Participants were grouped by PNES patients with a prior epilepsy diagnosis (PNES+ES) and PNES patients without a prior epilepsy diagnosis (PNES-ES). Our study design is illustrated in online supplemental eFigure 1.

### BZD prescriptions

Outpatient BZD prescriptions were evaluated in the year prior to the index PNES diagnosis and in the 30-day follow-up period (including the date of PNES diagnosis). BZDs were identified from prescription files, defined as the presence of >1 fill for a BZD. We selected 1-year prior to the index date as the start of our baseline observation window, as past studies have illustrated a particularly high burden of acute care utilisation in the 12 months preceding PNES diagnosis.^20 21^ BZD fills were also measured specifically in the 30 days before the index PNES diagnosis as a proxy for adults currently on BZD treatment. We examined BZD treatment in the 30-day follow-up period to allow for delays in follow-up visits, subsequent specialty care visits and/or delays in filling a prescription, particularly for those with current BZD use. We included the index date in the 30-day follow-up period to account for prescriptions that may have resulted directly from the PNES diagnosis. Given that tolerance can be developed within 1–2 weeks of BZD treatment,^22^ we used a supply of >7 days as a threshold to delineate short-term versus longer-term BZD prescriptions.

### Additional measures

We also examined demographics (age at the time of index event, sex) and clinical characteristics, including prior year psychiatric and non-psychiatric diagnoses commonly associated with both PNES and ES, healthcare use variables (eg, baseline inpatient admissions) and baseline psychotropic medication fills. Psychiatric characteristics included diagnoses of depression, anxiety disorder, psychotic disorders and substance-related disorders, among other disorders.

### Statistical analysis

First, we described the demographic and clinical characteristics of individuals newly diagnosed with PNES. Second, we calculated the prevalence of baseline BZD receipt, including fills in the year and 30 days prior to the index date (ie, new PNES diagnosis). Third, we estimated the prevalence of post-index BZD receipt in the 30-day follow-up period after the index PNES diagnosis. Fourth, we estimated the prevalence of post-index BZD receipt stratified by whether the individual had filled BZDs prior to the index PNES diagnosis. Fifth, recognising that people commonly receive BZDs for co-occurring ES, anxiety and sleep disorders, we conducted a series of secondary analyses: (1) We compared BZD receipt rates among people with and without baseline ES diagnoses, (2) we estimated BZD receipt rates among individuals without baseline anxiety and insomnia diagnoses. Finally, we used logistic regression to identify independent predictors of post-index BZD receipt, including demographic and clinical characteristics into the full model, computing variance inflation factors to evaluate for multicollinearity, we found no significant collinearity among all covariates using a threshold of less than 2.0.

Statistical analysis was performed using SAS V.9.4 from October 2023 through July 2024.

## Results

### Study sample and baseline characteristics

The sample of 20 848 privately insured adults who received a new diagnosis of PNES was primarily female (70.6%) with a median age of 42 years (table 1). Over one-quarter (n=5692; 27.3%) had an ES diagnosis in the year preceding their index diagnosis of PNES. Over three-quarters of the sample had a baseline non-substance use disorder psychiatric diagnosis (table 1). The most common baseline psychiatric diagnoses were anxiety disorders (56.6%) and depression (45.4%). Baseline diagnoses of post-traumatic stress disorder were comparatively less common (14.7%), as were personality disorders (3.8%). Pain-related diagnoses including musculoskeletal pain (43.4%) and headache/migraine (39.0%) were common, along with fatigue/malaise (35.6%). With regard to non-BZD medications, overall, 35.7% had >1 opioid prescription filled in the baseline year, with Nonsteroidal anti-inflammatory drugs (NSAIDs) (30.0%) and skeletal muscle relaxants (23.3%) also being common. One-fifth (18.6%) had at least one baseline script for antiseizure medication.

**Table 1:** Clinical characteristics of adults with a PNES diagnosis

	Total
	N=20 848 persons
Male sex/gender	6119 (29.4%)
Age, median (IQR)	42 (26–53)
Mental health characteristics, prior year	
Inpatient admission (excludes PNES diagnosis date)	
Inpatient psychiatric-related admission	1515 (7.3%)
Inpatient non-psychiatric-admissions	4087 (19.6%)
Any psychiatric diagnosis (excluding substance use disorder)	15 862 (76.1%)
Depression	9456 (45.4%)
Anxiety disorder	11 805 (56.6%)
Adjustment disorder	2736 (13.1%)
Acute stress disorder	383 (1.8%)
Attention-deficit hyperactivity disorder	1950 (9.4%)
Post-traumatic stress disorder	3060 (14.7%)
Schizophrenia	524 (2.5%)
Bipolar disorder	2338 (11.2%)
Personality disorder	789 (3.8%)
Other psychoses	899 (4.3%)
Other episodic mood disorder	1135 (5.4%)
Obsessive-compulsive disorder	629 (3.0%)
Conduct disorder	327 (1.6%)
Eating disorder	463 (2.2%)
Tic diagnosis	187 (0.9%)
Sleep disorder	4192 (20.1%)
Suicidal ideation	1203 (5.8%)
Tobacco dependence	2617 (12.6%)
Any substance use disorder	2895 (13.9%)
Psychotropic medication filled, prior year	
Antidepressant	11 418 (54.8%)
Stimulants	1793 (8.6%)
Hypnotic, other	1625 (7.8%)
Antipsychotic	3174 (15.2%)
Mood stabiliser, lithium	3485 (16.7%)
Hydroxyzine	2277 (10.9%)
Z-drug	1447 (6.9%)
Other medications filled, prior year	
Opioid	7437 (35.7%)
Gabapentin, pregabalin	3903 (18.7%)
Other anticonvulsant	3876 (18.6%)
Non-steroidal anti-inflammatory drugs	6262 (30.0%)
Beta-blocker	6760 (32.4%)
Skeletal muscle relaxant	4862 (23.3%)
Non-psychiatric clinical characteristics, prior year	
Sleep apnoea	2654 (12.7%)
Headache, migraine	8125 (39.0%)
Fatigue, malaise	7417 (35.6%)
Syncope, dizziness	5940 (28.5%)
Musculoskeletal pain (excluding low back)	9052 (43.4%)
Myalgia, fibromyalgia, myositis	2795 (13.4%)
Low back pain	4665 (22.4%)
Nervous system pain	3703 (17.8%)
Other, unspecified pain	991 (4.8%)
Muscle spasm	3764 (18.1%)
Poisoning, adverse drug effect	1248 (6.0%)

PNES, psychogenic non-epileptic seizures.

### Benzodiazepine prescribing characteristics

Overall, 33.1% received BZD treatment in the year before their PNES diagnosis, and 15.1% received BZD treatment in the month prior to PNES diagnosis (table 2). 17% filled both a BZD and opioid prescription in the prior year, and 3.5% received both in the 30 prior to PNES diagnosis.

**Table 2:** BZD prescription characteristics before and after new PNES diagnosis

	Total	
	N=20 848	
	Num. of people	% BZD pre
BZD prior to PNES diagnosis		
BZD prior year	6903	33.1
BZD prior 30 days	3149	15.1
**BZD post PNES diagnosis**	**Num. of people**	**% BZD post**
BZD in 30 days post	3777	18.1
Most common BZDs*		
Clonazepam	1421	33.0
Alprazolam	1119	26.0
Lorazepam	1042	24.2
Diazepam	470	10.9
Temazepam	114	2.6
Other	143	3.3
Days’ supply†		
Median (IQR)	30 (20–30)	
>7 days	3335	88.0
Stratified by prior BZD use		
No BZD filled in prior year	13 945	
BZD post 30 days	754	5.4
>1 BZD filled in prior year	6903	
BZD post 30 days	3023	43.8
>1 BZD filled, prior 30 days	3149	
BZD post 30 days	1981	62.9

* For individuals with multiple BZD prescriptions, each agent was counted.

† For individuals with multiple BZD prescriptions, days supply were summed.

BZD, benzodiazepine; PNES, psychogenic non-epileptic seizures.

Overall, 18.1% received BZD treatment in the month after a new PNES diagnosis, representing 43.8% of those with BZD prescription in the year prior to PNES. The most common BZDs received post-PNES diagnosis were clonazepam (33%), alprazolam (26%), lorazepam (24.2%) and diazepam (10.9%). The median days’ supply was 30 days (IQR=20–30). BZD use following a PNES diagnosis varied substantially by BZD receipt prior to the index PNES diagnosis. Among people without baseline BZD prescriptions, 5.4% (n=754) initiated BZD treatment during follow-up in the month post-PNES. In contrast, among those with baseline BZD prescriptions (n=6903), 43.8% (n=3023) filled BZDs in follow-up. In the subset of people with past-month BZD prescriptions, 62.9% filled BZDs in follow-up.

In our secondary analyses, we analysed patterns of BZD receipt after a new PNES diagnosis stratified by baseline diagnoses of ES, a common indication for BZD prescribing. Online supplemental eTable 1 illustrates that baseline BZD prescriptions were more common for those with baseline epilepsy diagnoses (41.3%) than among peers without baseline ES diagnoses (30.1%). Yet, rates of post-PNES BZD prescribing were similar among people with and without baseline ES diagnoses: for example, among people with past-month BZD prescriptions, 63.9% of people with ES received BZDs post-PNES, compared with 61.4% of peers without ES.

Finally, we conducted a secondary analysis that excluded individuals with baseline anxiety and sleep disorders (online supplemental eTable 2), another common indication for BZD receipt. The overall number of people without anxiety and sleep disorders receiving BZDs was relatively few, both before PNES (15.6% past-year; 6.0% past-month) and after PNES (8.9% 30 days post). Similar to primary analyses, the majority of people who were already on BZDs in the month prior to the PNES diagnosis received BZDs in the month after diagnosis. Even among people who had no baseline diagnoses for ES, anxiety and/or insomnia, 53.8% of past-month recipients of BZDs received BZDs in the month following PNES diagnosis.

### Predictors of benzodiazepine receipt

Table 3 depicts multivariable models illustrating the predictors of post-PNES BZD prescribing, including age, sex, baseline BZD receipt, baseline non-BZD use, past health services use and medical comorbidities in the model. With regards to demographic predictors, female sex was independently associated with increased odds of BZD prescribing (OR=1.16, 95% CI (1.04 to 1.28)) after a new PNES diagnosis, whereas younger age (18–29 years) was associated with lower odds compared with older peers (50–64 years) (OR=0.75 (95% CI 0.66 to 0.85)). Pre-PNES BZD treatment was strongly associated with post-PNES BZD prescribing. People with past-month BZD scripts had a 21-times greater odds of having a post-PNES BZD script (95% CI 18.84 to 23.61) than peers without past-year scripts. Anxiety disorders independently predicted post-PNES BZD receipt (OR=1.48, (95% CI 1.34 to 1.65)), we found a weaker association between BZDs filled in the 1 month after a PNES diagnosis and epilepsy (OR: 1.10, 95% CI 0.99 to 1.23) and sleep disorders (OR: 0.99, 95% CI 0.89 to 1.10).

**Table 3:** Independent predictors of benzodiazepine prescribing, full model

OR estimates			
Patient Characteristic	Point estimate	95% Wald	
		Confidence limits	
Female vs male sex	1.16	1.04	1.28
Ages 18–29 vs 50–64 years	0.75	0.66	0.85
Ages 30–49 vs 50–64 years	1.01	0.91	1.11
Benzodiazepine prescribing			
Past year BZD script (31-365 days prior) vs none in past year	5.27	4.72	5.88
Past month BZD script (1-30 days prior) vs none in past year	21.09	18.84	23.61
Mental health characteristics, prior year			
Inpatient admission (excludes PNES diagnosis date)			
Inpatient psychiatric admission	0.91	0.75	1.10
Inpatient non-psychiatric admissions	0.86	0.76	0.96
Any psychiatric diagnosis (excluding substance use disorder)			
Depression	0.96	0.87	1.06
Anxiety disorder	1.48	1.34	1.65
Adjustment disorder	0.96	0.85	1.08
Acute stress disorder	0.93	0.70	1.23
Attention-deficit hyperactivity disorder	0.95	0.79	1.13
Post-traumatic stress disorder	1.06	0.94	1.19
Schizophrenia	1.00	0.77	1.31
Bipolar disorder	1.23	1.07	1.42
Personality disorder	0.94	0.76	1.16
Other psychoses	1.03	0.84	1.28
Other episodic mood disorder	1.13	0.95	1.34
Obsessive-compulsive disorder	1.22	0.98	1.53
Conduct disorder	1.02	0.73	1.42
Eating disorder	0.88	0.67	1.16
Tic diagnosis	0.88	0.58	1.32
Sleep disorder	0.99	0.89	1.10
Suicidal ideation	0.82	0.67	1.01
Tobacco dependence	1.06	0.94	1.21
Any substance use disorder	1.05	0.93	1.20
Psychotropic medication (non-BZD) filled, prior year			
Antidepressant	1.14	1.03	1.27
Stimulants	1.10	0.93	1.31
Hypnotic, other	1.01	0.88	1.16
Antipsychotic	1.09	0.97	1.24
Mood stabiliser, lithium	1.10	0.98	1.23
Hydroxyzine	0.90	0.79	1.03
Z-drug	1.24	1.08	1.43
Other medications filled, prior year			
Opioid	1.27	1.15	1.40
Gabapentin, pregabalin	0.90	0.81	1.01
Other anticonvulsant	1.17	1.04	1.31
Non-steroidal anti-inflammatory drug	1.00	0.90	1.10
Beta-blocker	1.11	1.00	1.24
Skeletal muscle relaxant	1.16	1.04	1.29
Non-psychiatric clinical characteristics, prior year			
Epilepsy	1.10	0.99	1.23
Sleep apnoea	0.87	0.76	0.98
Headache, migraine	0.96	0.87	1.05
Fatigue, malaise	1.01	0.92	1.11
Syncope, dizziness	0.96	0.87	1.06
Musculoskeletal pain (excluding low back)	0.95	0.86	1.04
Myalgia, fibromyalgia, myositis	0.99	0.87	1.11
Low back pain	0.93	0.84	1.04
Nervous system pain	1.04	0.93	1.17
Other, unspecified pain	0.98	0.81	1.17
Muscle spasm	1.07	0.96	1.19
Poisoning, adverse drug effect	0.89	0.75	1.06

BZD, benzodiazepine.

## Discussion

To our knowledge, this is the first national study of BZD prescribing in a cohort of people with PNES, and our findings have important implications for informing future research efforts to improve care and treatment for PNES. Overall, in a nationwide sample of privately-insured adults, we observed that approximately one-third of those with a new PNES diagnosis had been prescribed a BZD in the year prior to their diagnosis. 18.1% received BZDs in the month following a PNES diagnosis, with a median days’ supply of 30 days, and clonazepam, alprazolam and lorazepam representing the most common BZDs prescribed after PNES diagnosis. While our data cannot precisely show why people were being prescribed BZDs, we found that nearly 90% received scripts exceeding 7 days, suggesting that few individuals were receiving BZDs purely for symptomatic treatment of acute PNES. While the potential for risks associated with continued BZD prescribing is potentially heightened by co-occurring opioid use (nearly 40% of the sample at baseline), the percentage receiving both opioids and BZDs was lower (17.3% in prior year; 3.5% in 30 days prior to PNES).

This study does not directly address whether correctly diagnosing PNES will reduce BZD use in the management of seizures; however, our data suggests that the deprescribing of BZDs after PNES diagnosis—among people already receiving BZDs is not the most common treatment course in current practice. Our data shows that BZD fills following a PNES diagnosis were heavily dependent on prior BZD use, a pattern that was even seen among people without common indications for BZDs (ie, diagnoses of epilepsy, anxiety and insomnia). Specifically, the majority of people who received BZDs in the month prior to PNES diagnosis remained on BZDs in the 1-month follow-up period. This practice may be at odds with standard clinical practice, which encourages the deprescribing of anticonvulsant medication in PNES. LaFrance and colleagues noted that, according to international surveys of clinicians in the USA, South America and Europe, ‘current standard medical care’ (treatment as usual) for PNES involves the tapering of antiseizure medications and the deferring of psychotropic medication initiation to a mental health specialist.^23^ Many neurologists and epileptologists may feel uncomfortable tapering BZDs, deferring to mental health professionals, a process that can be hindered by inadequate availability of collaborative care. It is possible that neurologists may not be the physicians prescribing the BZDs and may also feel uncomfortable initiating a taper. Additionally, community physicians may refer patients with suspected PNES to tertiary epilepsy centres for video electroencephalogram, another potential reason for deprescribing of BZDs to be delayed.

The high prevalence of co-occurring psychiatric disorders in the PNES cohort may mean these individuals experience anxiolytic and/or antidepressant benefits from BZDs. Poor quality sleep and anxiety are common problems in people with PNES.^24 25^ Historically, BZDs were commonly prescribed as a front-line long-term therapy for anxiety disorders and insomnia in the general population; while recent literature has raised concerns about the long-term risks of BZD treatment outweighing short-term mental health benefits,^26^ there is limited data to guide clinical practice on the use of BZDs to treat anxiety and sleep problems specifically in people with PNES. While a study of BZD prescribing in the immediate post-PNES period showed that BZD treatment did not reliably terminate seizure activity among people misdiagnosed with status epilepticus,^11^ there remains a dearth of studies investigating the use of BZDs for prophylactic treatment or acute management of PNES. Overall the risk/benefit ratio for both short-term and long-term use of BZDs in PNES remains poorly understood.

While most of our nationwide sample was not prescribed a BZD in the month after PNES diagnosis, single-site studies have suggested this practice may be common at certain institutions,^10^ suggesting there is heterogeneity in BZD prescribing patterns across different institutions and healthcare systems. Especially since BZD tapering among people prescribed for stable long-term treatment can be associated with anticipated harms,^27^ these results should not be taken as a call to rapidly deprescribe BZDs in people with PNES. While examining subsequent patient outcomes was outside the scope of this study, further work is needed to determine whether, and how, BZD treatment should be prescribed in this population with many psychiatric comorbidities and complex treatment needs, which could help inform treatment decisions.

There are several key limitations to consider. First, we are only able to capture PNES events that culminate in healthcare encounters; we cannot rule out the possibility of PNES being misdiagnosed and/or missing in these administrative data.^28^ Second, because the sample included adults with stable enrolment in private insurance, findings may not be generalisable to children and those with less stable insurance coverage.^29 30^ Third, our study only evaluates potential BZD prescribing during the 30 days following a new PNES diagnosis and may not capture more delayed prescribing changes that are, nevertheless, related to the incident PNES. Finally, we are unable to determine the validity of epilepsy diagnoses, and it is possible that some epilepsy diagnoses were re-evaluated in adults later presenting with PNES. Epilepsy diagnoses may be inaccurate in many cases, and it may be difficult to correct a misdiagnosis; studies found that 41% of people with PNES without ES were still prescribed antiseizure medications 4 years after diagnosis.^31^ In a systematic review of ICD-10 codes for ES, the positive predictive value of ES diagnoses was estimated to range from 62 to 90+%, with a specificity of 69.6%^32^; validation studies have not been done for ES codes using national claims data and thus we are unable to definitively deduce if the epilepsy diagnosis reflects a PNES with baseline epilepsy or potential misdiagnosis. Future research with enhanced clinical data is needed to untangle the diagnosing and misdiagnosing of epilepsy in patients newly diagnosed with PNES.

Despite these limitations, this study is strengthened by its use of multistate population-level data to evaluate potential unmet treatment needs among patients with PNES, a highly stigmatised and understudied condition. As evidence-based psychological and multimodal interventions are lacking,^33^ future research is needed to understand prescribing decisions and patient outcomes. Estimates on the use of BZD treatment in adults with PNES may help physicians and policymakers devise targeted intervention to address treatment gaps in people with PNES.

## Conclusion

PNES is a challenging condition to appropriately diagnose and treat. This analysis shows that BZD treatment is prevalent in people newly diagnosed with PNES. For the first time in a large, privately insured national sample of adults diagnosed with PNES, we described the patterns of BZD prescribing. Our results suggest there are a variety of treatment practices following a PNES diagnosis, including new BZD use, no BZD use and, among those already on BZD treatment, both stoppage and continuation of BZD treatment. New BZD initiation after PNES diagnosis was relatively uncommon (5.2–6.2%), suggesting clinical practice largely aligns with recommendations for BZD naive patients. However, this was not true for people with a history of BZD use, who likely continued BZDs after PNES. Overall*,* these results can contribute to future efforts to ultimately have clear treatment guidelines in this population. Because BZD withdrawal has clinical sequelae that may mimic both PNES and ES,^16 17^ our results suggest that clinicians may benefit from considering prior BZD exposure on the differential of potential diagnoses for patients being evaluated for PNES. Future investigation is needed to understand the risks/benefit ratio associated with BZD exposure in people with PNES.

## Supplementary material

10.1136/bmjno-2024-000767 online supplemental file 1

## Data Availability

Data may be obtained from a third party and are not publicly available.
