# Supplementary material for: Benzodiazepine receipt in adults with psychogenic non-epileptic seizures in the USA
Source: BMJ Neurol Open. 2024 Sep 18;6(2):e000767. doi: 10.1136/bmjno-2024-000767 (PMC11418548; doi:10.1136/bmjno-2024-000767)
Supplement: online supplemental file 1 [file bmjno-6-2-s001.docx]

**Online-Only Supplement**

**eFigure 1. Design Diagram**

**eTable 1. Stratified counts for the proportion of people with a BZD filled in the month following a PNES diagnosis by comorbid epilepsy diagnosis**

**eTable 2. BZD use prior to and post a new PNES diagnosis in those without an anxiety/insomnia diagnosis leading up to the new PNES diagnosis**

| **eFigure 1. Design Diagram** |
| --- |

The 1-year prior to PNES diagnosis represents the “baseline” period for covariate assessment, with the first diagnosis of PNES representing the index date (time=0).

PNES-ES

First Diagnosis of PNES

(“Index” Diagnosis)

1 year prior to PNES diagnosis

No diagnoses for ES in the

1 year before PNES diagnosis

BZD receipt assessed 1 month after PNES diagnosis

BZD receipt assessed 1 month prior to PNES diagnosis

BZD receipt assessed 1 year prior to PNES diagnosis

PNES+ES

First Diagnosis of PNES

(“Index” Diagnosis)

1 year prior to PNES diagnosis

>1 diagnoses for ES in the

1 year before PNES diagnosis

BZD receipt assessed 1 month after PNES diagnosis

BZD receipt assessed 1 month prior to PNES diagnosis

BZD receipt assessed 1 year prior to PNES diagnosis

| **eTable 1. BZD use prior to and in the month post a new PNES diagnosis in adults, stratified by comorbid epilepsy diagnosis** | | | | | | | | |
| --- | --- | --- | --- | --- | --- | --- | --- | --- |
|  | Total | | Epilepsy  (PNES+ES) | | No Epilepsy  (PNES-ES) | |  |  |
|  | N=20,848 | | N=5,692 persons | | N=15,156 persons | |  |  |
|  | Num. of People | % BZD pre | Num. of People | % BZD pre | Num. of People | % BZD pre |  |  |
| **BZD prior to PNES diagnosis** |  |  |  |  |  |  |  |  |
| BZD prior year | 6,903 | 33.1 | 2,348 | 41.3 | 4,555 | 30.1 |  |  |
| BZD prior 30 days | 3,149 | 15.1 | 1,130 | 19.9 | 2,019 | 13.3 |  |  |
|  |  |  |  |  |  |  |  |  |
| **BZD post PNES diagnosis** | Num. of People | % BZD post | Num. of People | % BZD post | Num. of People | % BZD post |  |  |
| BZD in 30 days post, overall | 3,777 | 18.1 | 1,282 | 22.5 | 2,495 | 16.5 |  |  |
|  |  |  |  |  |  |  |  |  |
| Stratified by prior BZD use |  |  |  |  |  |  |  |  |
| No BZD filled in prior year | 13,945 |  | 3,344 |  | 10,601 |  |  |  |
| BZD post 30 days | 754 | 5.4 | 206 | 6.2 | 548 | 5.2 |  |  |
| >1 BZD filled in prior year | 6,903 |  | 2,348 |  | 4,555 |  |  |  |
| BZD post 30 days | 3,023 | 43.8 | 1,076 | 45.8 | 1,947 | 42.7 |  |  |
| >1 BZD filled, prior 30 days | 3,149 |  | 1,130 |  | 2,019 |  |  |  |
| BZD post 30 days | 1,981 | 62.9 | 722 | 63.9 | 1,239 | 61.4 |  |  |

| **eTable 2. BZD use prior to and post a new PNES diagnosis in adults without an anxiety/insomnia diagnosis leading up to the new PNES diagnosis** |
| --- |

|  | No baseline anxiety or insomnia diagnosis | | No baseline epilepsy, anxiety, or insomnia diagnosis | |
| --- | --- | --- | --- | --- |
|  | N=7,915 | | N=5,896 persons | |
|  | Num. of People | % BZD pre | Num. of People | % BZD pre |
| **BZD prior to PNES diagnosis** |  |  |  |  |
| BZD prior year | 1,238 | 15.6 | 793 | 13.4 |
| BZD prior 30 days | 475 | 6.0 | 286 | 4.9 |
|  |  |  |  |  |
| **BZD post PNES diagnosis** | Num. of People | % BZD post | Num. of People | % BZD post |
| BZD in 30 days post overall | 707 | 8.9 | 461 | 7.8 |
|  |  |  |  |  |
| Stratified by prior BZD use |  |  |  |  |
| No BZD filled in prior year, | 6,677 |  | 5103 |  |
| BZD post 30 days | 268 | 4.0 | 198 | 3.9 |
| >1 BZD filled in prior year | 1,238 |  | 793 |  |
| BZD post 30 days | 439 | 35.5 | 263 | 33.2 |
| >1 BZD filled, prior 30 days | 475 |  | 286 |  |
| BZD post 30 days | 267 | 56.2 | 154 | 53.8 |
